# Detection of chronic wasting disease in feces and recto-anal mucosal associated lymphoid tissues with RT-QuIC in a naturally infected farmed white-tailed deer herd

**DOI:** 10.3389/fvets.2022.959555

**Published:** 2022-09-13

**Authors:** Deepanker Tewari, Melinda Fasnacht, Margaret Ritzman, Julia Livengood, Jessica Bower, Aaron Lehmkuhl, Tracy Nichols, Alex Hamberg, Kevin Brightbill, Davin Henderson

**Affiliations:** ^1^Pennsylvania Veterinary Laboratory, Harrisburg, PA, United States; ^2^United States Department of Agriculture, APHIS, Veterinary Services, National Veterinary Services Laboratories, Ames, IA, United States; ^3^Veterinary Services Cervid Health Program, United States Department of Agriculture, Animal and Plant Health Inspection Service, Fort Collins, CO, United States; ^4^Bureau of Animal Health, Pennsylvania Department of Agriculture, Harrisburg, PA, United States; ^5^CWD Evolution LLC, Fort Collins, CO, United States

**Keywords:** CWD (chronic wasting disease), deer, diagnostics, RT-QuIC, feces, RAMALT

## Abstract

Chronic wasting disease (CWD) is an infectious prion disease affecting the cervids, including white-tailed deer (WTD) (*Odocoileus virginianus*). CWD is typically diagnosed *postmortem* in farmed cervids by immunohistochemistry (IHC). Amplification-based detection methods are newer generation tests currently being evaluated to improve the detection of prion disease. In addition to improving sensitivity, *antemortem* detection by amplification assays is a focus for improving disease control and management. In this study, we evaluate the use of real-time quaking-induced conversion (RT-QuIC) to detect CWD in fecal and recto-anal mucosal-associated lymphoid tissue (RAMALT) samples from naturally infected farmed WTD herds at *postmortem*. We successfully detected the presence of CWD prions in WTD RAMALT with a specificity of 100% and a sensitivity of 85.7% (*n* = 71) and in feces with a specificity of 100% and a sensitivity of 60% (*n* = 69), utilizing RT-QuIC on samples collected *postmortem*. Seeding activity detected in RAMALT (15.3 ± 4.2%, *n* = 18) was much stronger than in feces (44.4 ± 4.2%, *n* = 15), as measured by cycle threshold (Ct) and rise in relative fluorescence in samples collected from the same WTD. Prion detection in the RAMALT (94.7%) and feces (70.5%) was highest when both obex and retropharyngeal lymph nodes (RPLNs) were positive for CWD *via* IHC. In the study group, we were also able to test prion protein gene variants and associated disease susceptibility. A majority of tested WTD were the CWD genotype (96 GG) and also harbored the highest percentage of positive animals (43.7%). The second highest population of WTD was the genotype 96 GS and had a CWD positivity rate of 37.5%. Each of these groups showed no difference in RAMALT or fecal detection of CWD.

## Introduction

Chronic wasting disease (CWD) is a prion disease of white-tailed deer (WTD) (*Odocoileus virginianus)* that results in the accumulation of abnormal prion protein (PrP^CWD^) in lymphoid and nervous tissue contributing to progressive disease and mortalities (1, 2). Official CWD surveillance in the United States is based on the detection of PrP^CWD^ in the retropharyngeal lymph nodes (RPLNs) and obex utilizing immunohistochemistry (IHC) (1–5). In farmed cervids, the development of more sensitive *antemortem* testing options that use easily accessible samples, such as recto-anal mucosa-associated lymphoid tissues (RAMALT) and feces, could provide valuable tools to help control CWD. Real-time quaking-induced conversion (RT-QuIC) has recently been shown to be a useful assay to detect abnormal prions, including those that cause CWD, in a wide variety of tissues and body fluids (1, 2, 4–8). The method is not yet approved for official disease surveillance in farmed cervids but has been reported to have 100% sensitivity and specificity when testing RPLN (8).

The promise of the next-generation tests such as RT-QuIC is potentially a very useful complement to current, harvest-dependent surveillance and warrants further investigation. In this study, we chose to assess the performance of RT-QuIC in a naturally infected herd of WTD using RAMALT and feces collected *postmortem*. The RT-QuIC assay offers some distinct advantages over other amplification assays as it does not generate infectious prions during *in vitro* amplification, and it is relatively easy to implement in a diagnostic setting (9). The RT-QuIC assay measures fluorescence from Thioflavin T (ThT) binding to amyloid fibrils of newly converted recombinant PrP^C^ seeded by the presence of PrP^CWD^ contained in the specimens. Alternating cycles of growth and fragmentation by shaking lead to logarithmic amplification of prion seeds. Here, we analyze not only diagnostic sensitivity and specificity for RT-QuIC in easy-to-collect *antemortem* samples (e.g., feces and RAMALT), but we also sought to understand genetic linkages between CWD infection, susceptibility, and CWD shedding status. The WTD herd tested was composed of multiple genotypes in the prion protein gene (*PRNP*) that has been shown to play a role in the CWD incubation period. Codons at locations 95, 96, 116, and 226 within the *PRNP* gene have previously been associated with increased survival time in CWD-infected WTD (10, 11). More recently, a whole genome analysis of genes that affect CWD has determined that there are other genomic factors, including single-nucleotide polymorphisms, that play a role in the CWD disease susceptibility (12).

This study uniquely combines the comparison of official CWD IHC diagnostic results from naturally exposed rather than experimentally inoculated farmed WTD with those of RT-QuIC that have applicability in *antemortem* testing, while also considering genetic polymorphisms in the *PRNP* gene.

## Materials and methods

### Ethical statement

Animals were euthanized following the AVMA guidance on euthanasia https://www.avma.org/sites/default/files/2020-02/Guidelines-on-Euthanasia-2020.pdf.

### Animals

The animals in the study were taken from a WTD herd located in Pennsylvania on approximately 20 acres of land. The WTD were kept in pens with common feeders and waterers. After the detection of CWD, upon approval by the United States Department and Pennsylvania Department of Agriculture, the herd was depopulated as a part of the CWD control program. Each animal was restrained, sedated, and then euthanized with a gunshot.

### Feces and tissue sampling

All samples were collected *postmortem* after euthanasia. Official tissue specimens (obex and RPLN) were collected in 10% formalin from the 254 animals that were eligible for testing. The CWD herd prevalence was found to be 26.3% with IHC conducted at the National Veterinary Services Laboratories. Ear tissues were available from 151 animals to help analyze *PRNP* gene variants and CWD infectivity and wherever possible, additional tissues were collected for RT-QuIC research. Research samples (fecal and RAMALT specimens) for RT-QuIC testing were collected as fresh tissues from only a subset due to convenience sampling.

Rectal cores were removed from each sampled WTD by cutting circumferentially around the anus and collecting approximately 10 cm of the distal rectum and anus. Fecal samples were removed from the rectum first upon arrival of rectal cores at the laboratory. The mucosal surface and feces remained intact in the rectum until first the feces, when present, were collected, and then RAMALT samples could be aseptically collected in the laboratory. For each fecal and RAMALT collection, new gloves, forceps, bench papers, and blades were used to collect in order to avoid cross-contamination. The rectal core was cut longitudinally, opened up, laid flat, and feces were removed anterior to the anus first and for RAMALT, a piece of rectal mucosa approximately 1 cm wide and 2.5–3 cm long was collected 1 cm anterior to the mucocutaneous junction. The samples were placed in individual whirlpaks. A total of 71 RAMALT and 69 fecal samples were available for RT-QuIC analysis for sensitivity and specificity determination from 85 animals (50 male and 35 female WTD with 47 between 1 and 3 years of age and with 37 between 4 and 6 years of age; Supplementary Table). Samples were stored at −80°C until they were prepared for RT-QuIC. Paired RAMALT and fecal samples were only available from 54 animals.

### Feces and RAMALT processing for RT-QuIC

Fecal homogenates were prepared at 10% of wet weight (0.3–1.5 g) using 1X phosphate-buffered saline (PBS). Feces were subjected to multitube vortexing for 15 min to break up the pellets. To further homogenize, the samples were placed on a rotator (Viral Antigens Inc.) for 35 min at room temperature before centrifugation at 14,000 × g for 30 min. A volume of 500 μl aliquots were stored at −80°C until the RT-QuIC test. RAMALT homogenates were prepared at 2% (wt/vol) in Biorad ELISA dilution buffer with 0.2 g of tissue and processed as per a tissue extraction protocol previously described using a Precellys (Bertim Inc.) extractor with the following settings: 4 cycles of 7,500 rpm at 30 s each. Processed samples were kept at −80°C until the RT-QuIC analysis was conducted (7).

### RT-QuIC assay

RT-QuIC was performed utilizing a truncated form of the Syrian hamster recombinant PrP substrate (SHrPrP) (residues 90 to 231) expressed and purified as described previously (7, 8, 13). The tissue homogenates were diluted in a ratio of 1:100 in 0.1% Sodium lauryl sulfate sodium dodecyl sulfate (SDS) buffer, and 2 μl of this resultant homogenate was added to 98 μl of RT-QuIC reaction buffer [320 mM NaCl, 20 mM Na_2_HPO_4_, and 1 mM Ethylene-diamine-tetraacetic acid (EDTA)], 1 mM ThT, and 0.1 mg/ml SHrPrP^C^ and incubated in a 96-well-plate, as described below on the reader. For feces, fecal homogenates were thawed and centrifuged at 14,000 × g for 30 min. The resultant pellet was resuspended in 100 μl of PBS and 7 μl of NaPTA (0.25 g sodium phosphotungstic acid and 0.125 g magnesium chloride hexahydrate in 5 ml of water; Sigma Inc.) and incubated for 90 min on a thermomixer, shaking at 1,400 rpm at 37°C (Eppendorf). The sample was then centrifuged for 30 min at 14,000 × g. The pellet was resuspended in 0.05% SDS in 1X PBS and diluted further in a ratio of 1:10. A volume of 2 μl of prepared sample dilution was added to 98 μl of RT-QuIC reaction buffer (320 mM NaCl, 20 mM Na2HPO4, and 1 mM EDTA), 1 mM ThT, and 0.1 mg/ml SHrPrPC and incubated in a 96-well, optical bottom plate (Nunc), as described below on the reader (14). Positive and negative controls were included with each run consisting of previously confirmed CWD IHC-positive lymph node at a 1:100 dilution and a no template control, respectively. Each specimen was tested in triplicate.

Plates were subjected to 250 cycles of shaking with cycles of 1 min of shaking (700 rpm, double orbital) and 1 min of rest with incubation at 42^**°**^C for RAMALT and 37^**°**^C for feces on a FLUOstar Omega fluorescence plate reader (BMG Labtech). ThT fluorescence readings (448 nm excitation and 482 nm emission, bottom read, 20 flashes per well at 4 mm setting) were taken following each 15-min cycle, using a gain setting of 1,700 with an incubation time of 62.5 h. Positive results were those that crossed a fluorescence threshold determined by the mean fluorescence of all sample baselines plus five standard deviations. The time to positivity was defined as the time at which a sample fluorescence emission crossed the cycle threshold (***C***_*****T*****_), and the time to reach maximum fluorescence (C ***max***) was also recorded. For feces or tissue specimens to be considered positive, at least two of the three wells for each tested specimen crossed the threshold. The analyses were performed using the MARS software (BMG Biotech), as previously described (7, 13, 14). Statistical data analyses, including test of significance, were determined using ***t***-tests. Test sensitivity and specificity were calculated with the following formulae: Sensitivity (Se) = True positive/True positive + False negative; Specificity (Sp) = True negative/True negative + False positive.

### Genotyping

DNA was extracted using the MagMAX CORE Nucleic Acid Purification kit (Applied Biosystems) on ear notch specimens collected using the Tissue Sampling Unit (Allflex) following the manufacturer's instructions. The prion protein gene (*PRNP*) was amplified using Platinum 2X Universal Mix (Invitrogen) with 0.4 μM of each of primers CWD-13 (5′-TTTTGCAGATAAGTCATGGTGAAA3′) and CWD-LA (5′AGAAGATAATGAAAACAGGAAGGTTGC-3′). PCR conditions were as follows: 95°C for 5 min, 10 cycles of denaturation at 95°C for 45 s, annealing at 58°C for 45 s, and extension at 72°C for 90 s, followed by 35 cycles of 95°C for 45 s, 57°C for 45 s, and 72°C for 90 s with a final extension at 72°C for 5 min, as previously described (10). PCR products were purified (Qiagen) and sequenced using amplification primers with Sanger sequencing, as previously described (Eurofins) (15). All sequences were individually analyzed for conflicts and secondary peaks and aligned to the *O. virginianus* reference sequence, AF 156185 (GenBank), considering only functional genes but not the pseudogene. The nucleotides and corresponding amino acid polymorphisms at codons 95 (Glutamine Q or Histidine H), 96 (Glycine G or Serine S), 116 (Alanine A or Glycine G), and 226 (Glutamine Q or Lysine K) were evaluated.

### IHC

IHC testing was conducted at NVSL, as previously described (5). Briefly, formalin-fixed tissues (medial RPLN and obex) were processed and embedded in paraffin. Sections were cut at 5 μm, and immunohistochemical staining was performed following the USDA CWD test protocol using an automated stainer and monoclonal F99 anti-prion antibody. A section of positive lymph node and obex and negative lymph node was included as a control with every run. Tissues were considered positive if staining of the appropriate character and anatomic location was observed. Tissues were considered negative if IHC staining of the appropriate character and anatomic location was not observed. Lymph nodes with 5 or fewer follicles in the entire section were considered insufficient to make a negative determination; there was no minimum number of lymphoid follicles necessary to make a positive determination.

## Results

### RAMALT sensitivity and specificity in RT-QuIC detection

RT-QuIC analysis of WTD RAMALT specimens showed an overall sensitivity of 85.7% and a specificity of 100% for PrP^CWD^ detection (*n* = 71) (Table 1A). RT-QuIC failed to detect CWD in the RAMALT of three WTD determined to be CWD-positive by IHC. Of those, one animal was IHC-positive for CWD in both the obex and the RPLN, suggesting this animal was in a later stage of infection; and the other two were IHC CWD-positive only in the RPLN, suggesting that these WTD were in an early stage of CWD (**Table 2**). RT-QuIC sensitivity in RAMALT was dependent upon the stage of disease in the WTD with RT-QuIC, correctly identifying 94.7% of animals were positive in both the obex and RPLN and 75% of animals were positive only in the RPLN (**Table 1C**). None of the WTD were RT-QuIC-positive if CWD was not detected by IHC (n = 50; Table 1B).

**Table 1A:** Sensitivity and specificity of rectal mucosal-associated lymphoid tissue for PrP^Sc^ detection with RT-QuIC in white-tailed deer.

**Specimen**	**CWD Status**	**CWD Detected**	**CWD Not Detected**	
**RAMALT**	CWD(+)	18 (TP)	0 (FP)	Sensitivity 85.7%
**RT-QuIC**	CWD(-)	3 (FN)	50 (TN)	Specificity 100%
**Feces**	CWD(+)	15 (TP)	0 (FP)	Sensitivity 60%
**RT-QuIC**	CWD(-)	10 (FN)	44 (TN)	Specificity 100%

TP, True positive; FP, False positive; FN, False negatives; TN, true negative.

RAMALT n = 71; Feces n = 69.

**Table 1B:** Sensitivity and Specificity of feces for PrP^*Sc*^ detection with RT-QuIC in white-tailed deer.

**Specimen**	**CWD Status**	**CWD Detected**	**CWD Not Detected**	
**RAMALT**	CWD(+)	18 (TP)	0 (FP)	Sensitivity 85.7%
**RT-QuIC**	CWD(-)	3 (FN)	50 (TN)	Specificity 100%
**Feces**	CWD(+)	15 (TP)	0 (FP)	Sensitivity 60%
**RT-QuIC**	CWD(-)	10 (FN)	44 (TN)	Specificity 100%

TP, True positive; FP, False positive; FN, False negatives; TN, true negative.

RAMALT n = 71; Feces n = 69.

### Feces sensitivity and specificity for RT-QuIC detection of CWD

Analysis of WTD fecal specimens showed an overall sensitivity of 60% and a specificity of 100% (*n* = 69) (Table 1A). Among 17 specimens tested positive for CWD in both RPLN and brain by IHC, 12 specimens tested positive by RT-QuIC with a 70.5% detection rate (Table 1C). However, in eight specimens from animals where CWD was detected only in RPLN by IHC, two specimens tested positive by RT-QuIC with a 25% detection rate (Table 1D). For paired RAMALT and feces collected from the same WTD, the detection rate with RT-QuIC was at 64.7% for feces when CWD prions were detected by IHC (11/17) (Table 2 and Supplementary Table).

**Table 1C:** CWD detection with RT QuIC in RAMALT and feces for white-tailed deer with prion detection CWD (+) in both lymph node and obex with immunohistochemistry.

**RAMALT**	CWD(+)	18/19	94.7%
**Feces**	CWD(+)	12/17	70.5%

**Table 1D:** CWD detection with RT QuIC in RAMALT and feces for white-tailed deer with prion detection CWD (+) in only lymph node with immunohistochemistry.

**RAMALT**	CWD(+)	6/8	75%
**Feces**	CWD(+)	2/8	25 %

**Table 2:** Rectal mucosa-associated lymphoid tissue (RAMALT) and feces RT-QuIC reactivity in deer showing prion protein detection with immunohistochemistry (IHC) collected from the same white-tailed deer (***n*** = 54).

**CWD IHC positive**	**RT QuIC RAMALT positive**	**RT QuIC RAMALT negative**	**Feces positive**	**Feces negative**
RPLN and Obex (*n* = 11)	10	1^a^	9	2^b^
RPLN only (*n* = 6)	4	2^c^	2	4^d^

Negative by all methods *n* = 37.

^a^GG genotype; 5years.

^a, b^GS genotype; 4years; 1 animal RAMALT positive.

^c^GS genotypes;6 and 2 years.

^c, d^GG genotype; 5 and 6 years.

### CWD detection by IHC among WTD with different *PRNP* genotypes

In the depopulated herd, a majority (74.2%) of WTD analyzed for prion protein genotypes had the codon 96 GG genotype, and 43.7% of those WTD were CWD-positive (Table 3). In addition, more than one-third (37.5%, 9/24) of the codon 96 GS WTD were positive for CWD (Table 3). A single 5-year-old codon 96 SS WTD was present in the herd and tested positive for CWD (Table 3). The heterozygous codon 95 HQ genotype represented about 8% (two WTD were 95 HQ and 96 GS in addition to 11 HQ, GG) of the herd and one of those, a 6-year-old codon 95 HQ and 96 GS WTD, tested positive for CWD (Table 3). A single WTD homozygous for 95 HH genotype was also observed with no detection of CWD. All WTD analyzed had AAQQ genotype at codons 116 and 226 and showed no heterogeneity.

**Table 3:** Detection of CWD in animals with immunohistochemistry (IHC) in white-tailed deer with different genotypes.

**Number of Animals**	**Conv**.	**Codon 95**	**Codon 96**	**CWD Detection (n)**	**Tissue Detection (IHC)** **(n)**		**Genotype Frequency**	**CWD detection within each genotype**
	Name*				**RPLN & Obex**	**RPLN**	**(n=151)**	
112	96GG	QQ	GG	49	38	11	74.2%	43.7%
24	96GS	QQ	**GS**	9	5	4	15.9%	37.5%
11	95HQ	**HQ**	GG	0	0	0	7.3%	0.0%
2	95HQ 96GS	**HQ**	**GS**	1	0	1	1.3%	50%
1	96SS	QQ	**SS**	1	0	1	0.7%	100%
1	95HH	**HH**	QQ	0	0	0	0.7%	0.0%

*Codons 116 and 226 were homogeneous as AAQQ.

Heterozygous codons 95 and 96 are denoted in bold.

### CWD detection with RT-QuIC and relationship of Ct values and maximum fluorescence in RAMALT and feces

**Figure 1:**
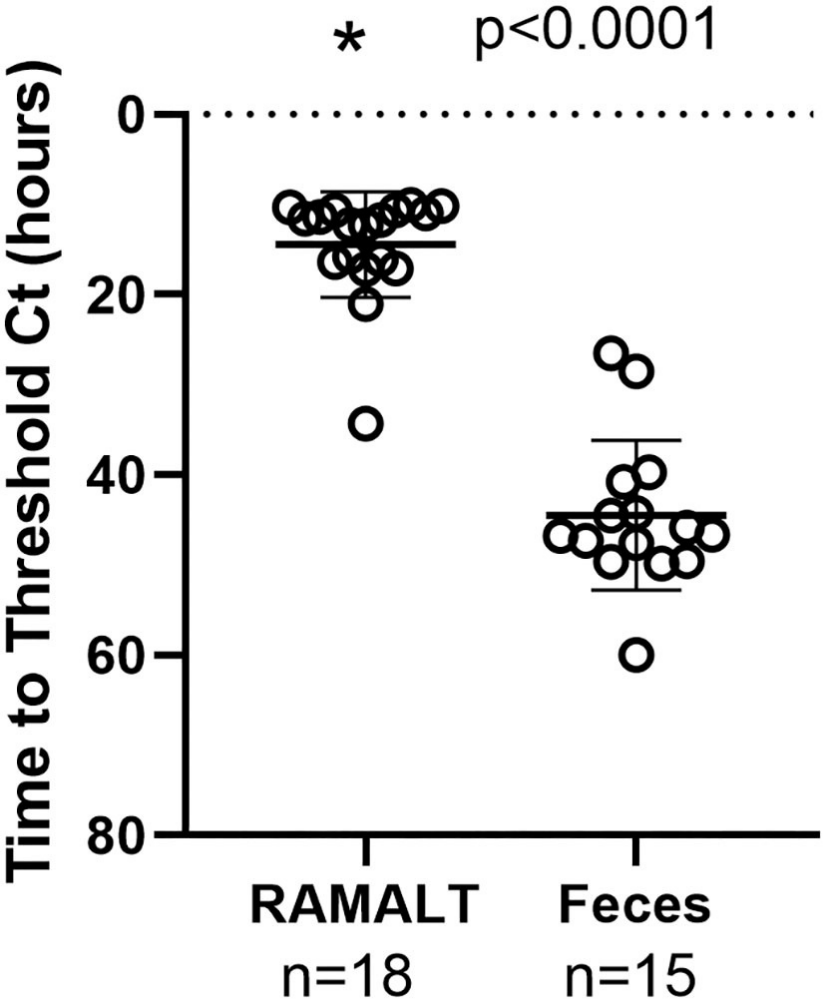
RT-QuIC testing of white-tailed deer RAMALT and feces and time to threshold (Ct) relationship. RAMALT (*n* = 18) has a significantly lower Ct value compared to fecal samples (*n* = 15) (*p* < 0.0001). The * symbol indicates Ct value.

**Figure 2:**
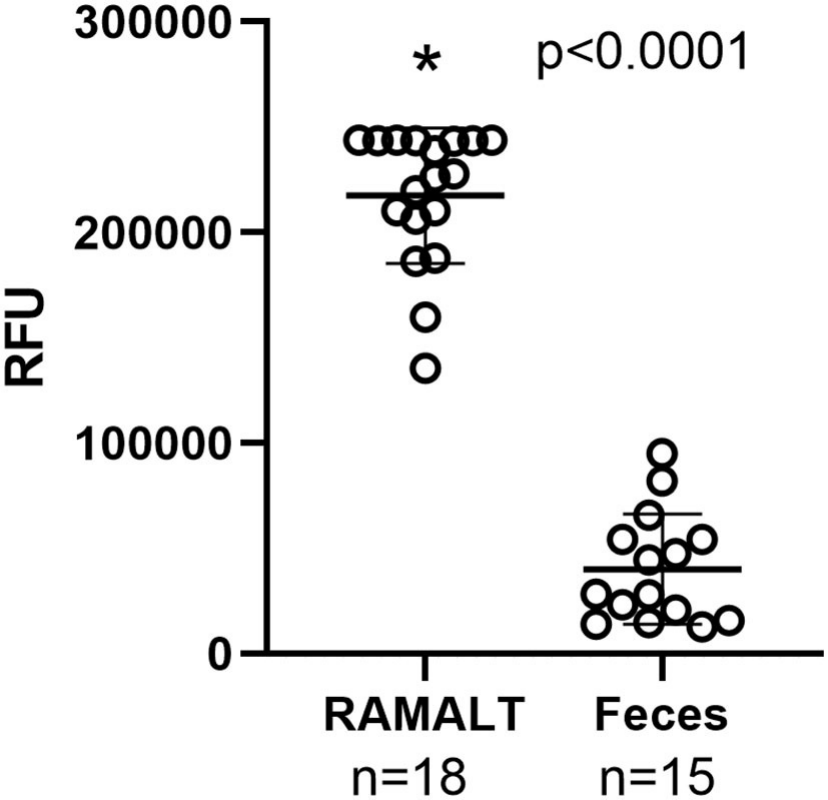
RT-QuIC testing of RAMALT and feces and relationship to fluorescence. RAMALT samples (*n* = 18) have significantly higher relative fluorescent units (RFUs) compared to white-tailed deer fecal samples (*n* = 15) (*p* < 0.0001). The * symbol indicates RFU value.

**Figure 3:**
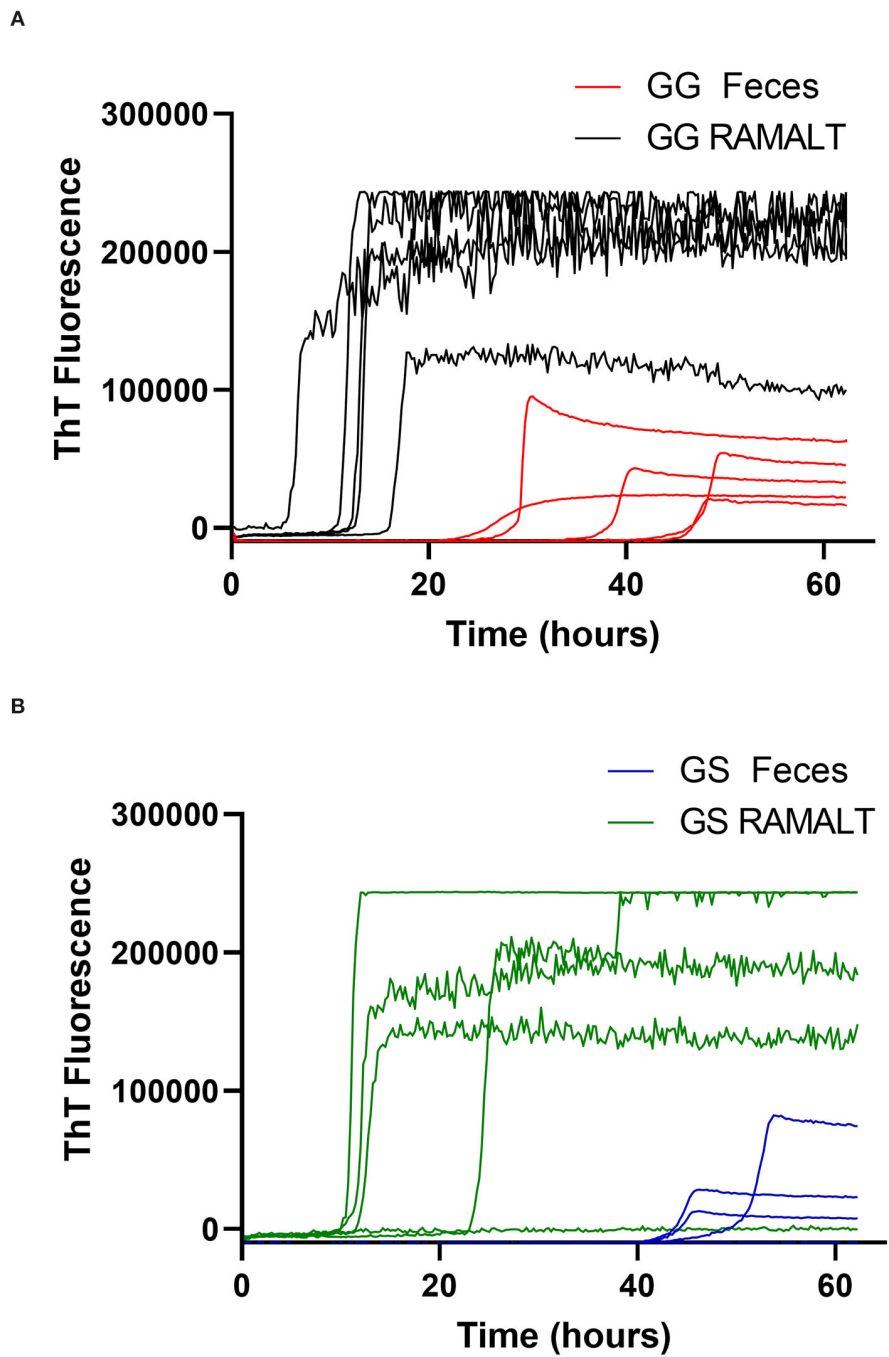
White-tailed deer genotype influence on RT-QuIC reaction kinetics. **(A,B)** Raw fluorescence traces for 96 GG and 96 GS deer comparing RAMALT fluorescence values and time to threshold to fecal values.

Cycle threshold (Ct) has been shown to be directly correlated to the seeding ability of a specimen and the amount of CWD prion in a specimen (11). RAMALT tissue showed significantly (*p* < 0.0001) lower Ct values compared to feces, suggesting higher levels of CWD are found in RAMALT (Figure 1). Seeding capacity detected in RAMALT (15.3 ± 4.2%, *n* = 18) was much stronger than in feces (44.4 ± 4.2%, *n* = 15), as measured by Ct. Relative fluorescence units (RFUs) were inversely correlated with Ct; a low Ct corresponded with high RFU, indicating stronger detection of CWD in RAMALT tissues (Figures 2, 3). RAMALT tissues had significantly higher RFU compared to fecal samples (*p* < 0.0001) (Figures 2, 3).

### CWD detection comparison among various *PRNP* genotypes with RT-QuIC to IHC detection in obex and RPLN

**Figure 4:**
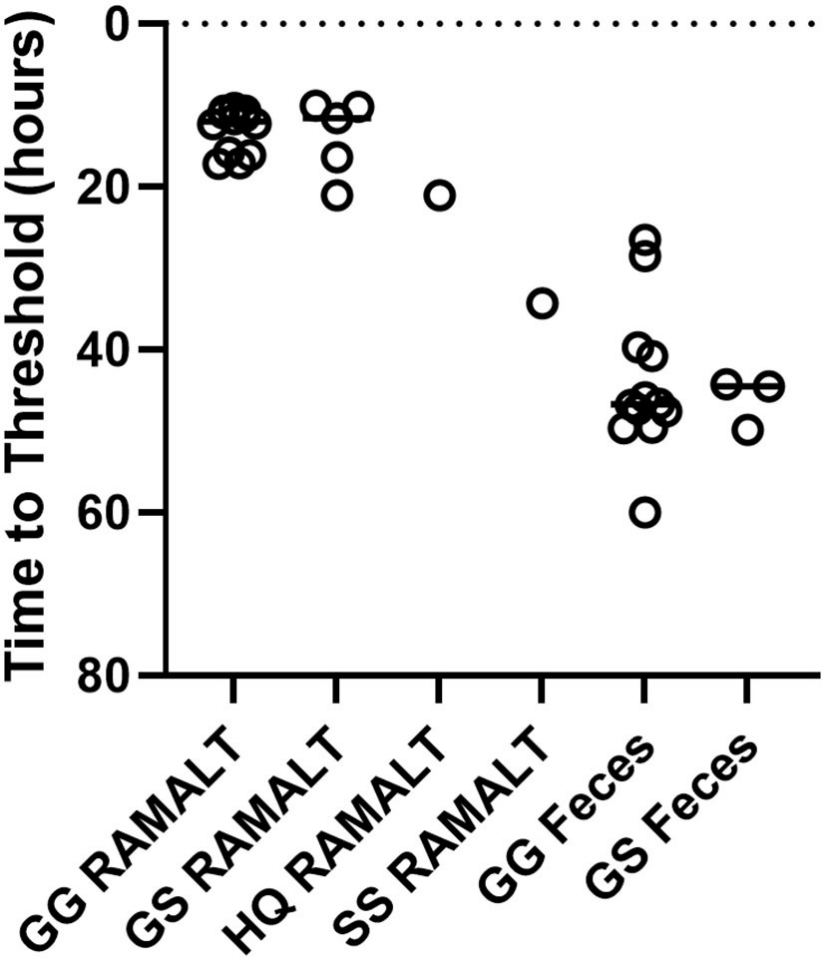
Genotype variation and Ct values. RT-QuIC testing of RAMALT and feces for CWD-infected white-tailed deer with different genotypes. No significant difference between 96 GG (*n* = 12) and 96 GS (*n* = 5 RAMALT and 3 feces) in time to threshold for RAMALT or feces was found. Not enough data were present to statistically analyze other genotypes.

One CWD-positive WTD with heterozygosity at codon 95 HQ and 96 GS was positive for CWD by RT-QuIC in the RAMALT with a Ct value of 21.8 and was not detected in the feces. Interestingly, this WTD was only IHC CWD-positive in RPLN but was not detected in the obex. A total of 11 other codon 95 HQ WTD had no detection of CWD by RT-QuIC or by IHC (Supplementary Table). No differences were observed between Ct values of feces from WTD with codon 96 GG and 96 GS (Figure 4) but other genotypes (e.g., 95 HQ and GS or 96 SS) showed a lower seeding activity; however, more CWD-positive samples from WTD with these rare polymorphisms will need to be statistically compared. There was no statistical difference between the detection of CWD in the feces of 96 GG or 96 GS WTD (Figure 4 and Supplementary Table). With HQ WTD, feces did not show any prions or seeding activity as detected by RT-QuIC, but we could not ascertain the same for 96 SS WTD due to feces unavailability. Out of eleven WTD that were CWD-positive by IHC in both obex and RPLN, ten were RAMALT-positive with a missed detection by RT-QuIC in only one WTD (GG genotype, age 5 years); and nine had CWD detected in feces. Two of the WTD where CWD was not detected with RT-QuIC, one WTD was the same as the one that missed detection in RAMALT having the GG genotype, and age 5 years; the second was a GS, age 4 years (Table 2). In addition, of the six WTD that showed CWD detection in RPLN only, four showed detections with RT-QuIC in RAMALT and missed prion detection in two animals having GS genotype (ages 6 and 2 years). While using feces for these six CWD-positive, RT-QuIC showed detections only in two animals. CWD was not detected in four animals. Two missed animals were the same as also missed with RAMALT RT-QuIC. The other two WTD that missed fecal detection, although were positive by RAMALT RT-QuIC, were both of GG genotype, with ages 5 and 6 years (Table 2 and Supplementary Table).

## Discussion

Improved *antemortem* detection tools for prion disease are desirable to help develop a successful CWD control program to address the continually expanding footprint of the disease (16). This is the first report of RT-QuIC testing in a naturally exposed and infected WTD herd with CWD where samples that can be taken from live animals (RAMALT and feces) were compared to *postmortem* results. This study also provides a deeper insight into WTD infections and susceptibility of WTD with certain genotypes in a naturally infected WTD herd in a confined setting encountering significant CWD exposure with high herd prevalence. We report the application of RT-QuIC on two WTD specimen types, RAMALT and feces, which are amenable to *antemortem* testing. In this study, sensitivity for RAMALT and feces was reported to be 85.7 and 60%, respectively; and specificity was 100% for both sample types. In spite of sampling being conducted *postmortem*, we have tried to approximate the sample size amount and location that simulate an *antemortem* RAMALT collection. RAMALT samples were taken from the same area and were of the same size as would be in a live animal sampling and fecal sampling shortly after death is nearly identical to *antemortem* sampling. Samples of this nature are very difficult to obtain and even with the limitations of *postmortem* sampling, this study gives us an insight into RT-QuIC application and what could be expected using the technique as an *antemortem* test for CWD detection. The application of RT-QuIC CWD detection in deer has been previously demonstrated in lymph nodes and in various other tissues, excreta, and skin of deer (6, 7, 13, 17, 18). Successful RT-QuIC detection of CWD in RAMALT from naturally infected elk has been shown to have a detection level between 70 and 90% (6, 19). With detection near 80% in a readily accessible tissue such as RAMALT, RT-QuIC testing of live animals may be a useful screening tool in farmed cervid operations or in selected natural settings (3, 5, 6, 20). RAMALT tissue is not the first tissue of the alimentary tract of deer shown to be CWD-positive; however, by using a more sensitive test such as RT-QuIC, the use of RAMALT for CWD diagnosis is continuing to show promise for disease management (21, 22).

In contrast, the detection of prions in feces with RT-QuIC, or any other amplification-based method, has been challenging. Despite inherently low levels of prions being shed in excreta compared to tissues, RT-QuIC has consistently demonstrated the presence of CWD in experimentally and naturally infected deer (23–25). Our data suggest that fecal samples that test positive for CWD in RT-QuIC can be useful for finding WTD that are in a later or shedding stage of CWD and may be more likely to spread CWD through increased shedding of CWD. This study is also the first documented comparison of detection of CWD in feces and RAMALT from the same WTD, which gives us a better understanding of the relationship between CWD detection in RAMALT and fecal prion shedding. The source of prion shedding in feces remains unknown but is likely a combination of routes from multiple cell types and origins.

In this study, we showed that RAMALT had a stronger seeding ability in RT-QuIC compared to feces as measured by Ct/Cmax and RFU, but was comparable with Ct, Cmax, and RFU observed earlier with RPLN (7). Our *postmortem* collection of feces was identical to methods used for live animal collection, and we anticipate that collection directly from live animals should demonstrate similar results and is being undertaken with other likely infected herds. A previously published study does not indicate that collection from the ground has a measurable inhibitory effect compared to a direct live animal collection but further studies that are needed to elucidate the effect of contamination on seeding capacity are also in progress (14). Testing feces as an *antemortem* specimen requires balancing both sensitivity and specificity in RT-QuIC testing parameters. Our preliminary studies had indicated using 40°C incubation as compared to 37°C increased sensitivity but also decreased specificity (Supplementary Table). After the initial validation work, all experiments, thereafter, were conducted with incubations at 37°C with sensitivity of 60% while maintaining specificity of 100%. Further improvement of prion detection sensitivity in feces with RT-QuIC is still a desirable goal.

Interestingly, we observed no seeding activity in three RAMALT samples with RT-QuIC that showed the presence of CWD prions with IHC testing. Two specimens tested positive only in RPLN by IHC, which indicated they were in an early stage of disease (22). However, one specimen was both brain and lymph node IHC-positive but remained RAMALT-negative with RT-QuIC even upon retesting. We believe this situation is similar to other cervids and sheep, where RAMALT positivity can sometimes lag behind other tissues or may be due to non-uniform prion distribution in the WTD or sample. It could also be due to sampling where a suboptimal section of the RAMALT was obtained containing no or few lymphoid follicles. Although RAMALT is not the earliest tissue to harbor CWD, it appears to balance the line between a relatively easy-to-collect sample with sensitivity high enough that *antemortem* RT-QuIC detection can be an important tool in CWD detection. Other samples such as tonsil would likely have higher sensitivity but the need for special tools for tissue collection and animal handling renders tonsil and other not easily accessible tissues less useful to be considered as an appropriate tissue for *antemortem* testing.

Multiple polymorphisms in the *PRNP* gene coding for the prion protein in WTD have been linked with increased incubation time for CWD (26, 27). Polymorphisms at positions 95, 96, 116, and 226 have all shown some evidence of delay in disease progression in a multitude of studies (6, 9, 10, 22, 23, 28). However, for CWD, disease resistance is not absolute (29). We and others have found animals with genotypes GS, SS, or HQ to be infected, pointing to likely additional genetic determinants behind disease susceptibility or resistance (9, 10, 13). CWD disease progression has been reported to be a heritable and polygenic trait (12). Even though our study qualitatively showed that 96 SS- and 95 HQ-infected WTD had lower levels of CWD as judged by Ct values derived from seeding activity, comparing more WTD with these less common genotypes to herd controls of more common WTD genotypes will be useful to determine if there is less accumulation of CWD prions in the tissues, including less shedding.

In summary, the sampling of RAMALT tissue combined with RT-QuIC testing appears to be a relatively sensitive, easy-to-perform assay to determine the CWD status of live animals.

While the RT-QuIC fecal detection data are promising, further optimization will be needed to determine if it can be helpful in identifying infected herds without individual animal testing.

## Author's note

CWD is a prion disease that affects the members of the family Cervidae and is unique in that it is the only prion disease to affect wild and free-ranging animals. CWD infection has spread through a combination of anthropomorphic events and the natural free range of animals to 30 US States and 3 Canadian Provinces. New cases of CWD have also been discovered in Norway, Finland, and Sweden. In spite of the concerted effort, CWD continues to manifest in new territory each year. Many previous studies on CWD have used point source inoculation of CWD in a laboratory setting to determine disease course, tissue distribution, and potential tissues suitable for live animal testing. Here, we examined WTD that have acquired CWD infection in a more natural setting as opposed to being experimentally infected within a farmed cervid environment. We explored the suitability of using RAMALT and feces collected from WTD at *postmortem* by RT-QuIC with implications that these samples, if they show similar sensitivity and specificity, can help improve *antemortem* testing in the future. Moreover, due to the depopulation of the herd, we were able to compare our test directly with *postmortem* official testing performed at NVSL. We achieved a sensitivity of 85.7% with RAMALT tissue and 60% with feces. Specificity for RAMALT tissue was 100% but has previously been an issue with fecal samples. Revision of the testing protocol for feces produced a specificity of 100%. This study provides key insight into the utility of RT-QuIC and shows future possibilities of evaluating CWD with sample collection that can be carried out in live animals and can become an important tool to better monitor CWD infections in farmed settings.

## Data availability statement

The original contributions presented in the study are included in the article/Supplementary material, further inquiries can be directed to the corresponding author/s.

## Ethics statement

Ethical review and approval was not required for the animal study because this was a study conducted for improving animal welfare by State Department of Agri and USDA for CWD disease control and authorized and approved by State Animal Health Official. Animals were euthanized as per AVMA guidance.

## Author contributions

DT, DH, KB, AH, and TN conceptualized the study. AL contributed to all IHC work and critical review, TN, KB, and AH contributed research samples and offered critical review. MF, MR, JB, and JL conducted the lab tests and analyses. DH and DT finalized the manuscript and analyzed outcomes and future scope. All authors contributed to the article and approved the submitted version.

## Conflict of interest

Author DH was employed by CWD Evolution LLC. The remaining authors declare that the research was conducted in the absence of any commercial or financial relationships that could be construed as a potential conflict of interest.

## Publisher's note

All claims expressed in this article are solely those of the authors and do not necessarily represent those of their affiliated organizations, or those of the publisher, the editors and the reviewers. Any product that may be evaluated in this article, or claim that may be made by its manufacturer, is not guaranteed or endorsed by the publisher.
